# Instability and results after non-operative treatment of large anterior glenoid rim fractures: is there a correlation between fragment size or displacement and recurrence?

**DOI:** 10.1007/s00402-021-04020-w

**Published:** 2021-08-02

**Authors:** Matthias Königshausen, Simon Pätzholz, Marlon Coulibaly, Volkmar Nicolas, Marc Vandemeulebroecke, Thomas Armin Schildhauer, Dominik Seybold

**Affiliations:** 1 Department of General and Trauma Surgery, University Bergmannsheil Bochum, Ruhr-University Bochum, Bürkle-de-la-Camp-Platz 1, 44789 Bochum, Germany; 2 Department of Radiology and Interventional Radiology, University Bergmannsheil Bochum, Ruhr-University Bochum, Bürkle-de-la-Camp-Platz 1, 44789 Bochum, Germany; 3 Biostatistical Sciences and Pharmacometrics, Novartis Pharma AG, Klybeckstrasse, 4057 Basel, Switzerland

**Keywords:** Glenoid rim fracture, Bony bankart lesion, Instability, Glenoid fracture, Shoulder dislocation

## Abstract

**Introduction:**

There is little data available on non-operative treatment of anterior glenoid rim fractures (GRF). Nothing is known about fracture size and displacement in comparison to clinical outcomes and instability in a mainly middle-aged patient population. The aim of this study was to demonstrate the results of non-operative treatment in anterior glenoid rim fractures with the special focus on potential instability/recurrence.

**Methods:**

The inclusion criteria were non-operatively treated anterior GRF of at least ≥ 5 mm width using the age- and gender-matched Constant/Murley score (a.-/g.-CMS) and the Western Ontario Instability Index (WOSI). Radiographic parameters (fracture morphology, displacement, major tuberosity fractures and Hill–Sachs lesion using initial CT and radiographs) and the proportion of the fractured glenoid were detected (2D-CT-circle-method) and osteoarthritis (A.P. and axial radiographs) was classified according to Samilson/Prieto. Proportion of fractured glenoid and medial displacement were correlated with the recurrence rate and the clinical scores.

**Results:**

*N* = 36 patients could be followed-up after a mean of 4.4 years [12–140 month, average age: 58 (± 13, 33–86) years]. The a.-/g.-CMS was 93 (± 11, 61–100) points, and the WOSI was 81% (± 22%, 35–100%) on average. The mean intraarticular displacement was 4 mm (± 3 mm; 0–14 mm). The 2D-circle-method showed a mean glenoid fracture involvement of 21% (± 11, 10–52%). Two cases of frozen shoulders and one case with biceps pathology were associated with the trauma. Within the followed-up patient group re-instability has occurred in *n* = 2 patients (6%) within the first two weeks after trauma. Osteoarthritis was found in *n* = 11 cases. There was no correlation between the scores and the fracture size/displacement [(a.-/g.-CMS vs. displacement: *r* = − 0.08; *p* = 0.6; vs. size: *r* = − 0.29; *p* = 0.2); (WOSI vs. displacement: *r* = − 0.14; *p* = 0.4; vs. size: *r* = − 0.37; *p* = 0.06)], but very large (≥ 21%) fractures with displacement ≥ 4 mm showed slightly worse results without significant difference (a.-/g.-CMS *p* = 0.2; WOSI *p* = 0.2). The apprehension test was negative in all patients at final follow-up.

**Conclusion:**

Non-operative treatment of anterior GRF was associated with overall good results within a mainly middle-aged larger patient group. Re-instability is rare and is not associated with fragment size but can occur in the first weeks after trauma. Size and dislocation of the fracture is not a criterion for the prognosis of potential instability.

**Level of evidence:**

Level IV, retrospective case series.

## Introduction

Large anterior glenoid rim fractures (GRF) are often associated with shoulder dislocations and must be differentiated from osteochondral avulsion lesions of the anterior glenoid rim (“chip fractures”) that consist only of a small cortical fragment [14, 21]. According to the classification of Ideberg et al., large GRF correspond to type 1B (> 5 mm), while “chip fractures” correspond to small bony bankart lesions of the glenoid rim (1A, < 5 mm) [14]. The anterior GRF is mainly the injury of middle-aged patients (> 40 years or even > 50 years) as demonstrated by several authors [14, 18, 21, 24, 27, 32], while smaller glenoid rim lesions (chip fractures) usually concern mainly younger individuals and may lead to anterior glenoid erosions [10, 34]. Next to shoulder dislocations, a further presumed trauma mechanism is impaction of the humeral head (HH) on the anterior glenoid rim without dislocation of the HH [9].

Several studies have reported reinstability or recurrence after non-operative treatment of primary shoulder dislocation focused on chip fractures or reported a mixed cohort without a clear definition between chip fracture and large GRF without defect quantification by CT scan [11, 28–30].

Therapy for large GRF is still controversial within the literature. Most studies of large GRF address operative (open or arthroscopic) treatment [3, 4, 20, 24, 26, 27, 32, 33], whereas two different study centers demonstrated good functional results after non-operative treatment with only one case of re-instability but without relevant clinical osteoarthritis (OA) [18, 21, 38]. However, clinical outcomes of non-operatively treated larger patient groups and the influence of size and dislocation of the fragment on recurrence after non-operative treatment has never been investigated. Thus, the aim of this study was to answer the following questions:

1. What was the outcome after non-operative treatment of anterior GRF within a larger patient group?
2. Is there a correlation between radiologically measured fragment size and displacement with clinically detectable instability or recurrence?
3. Are biomechanical tests of anterior chronic glenoid bone loss within the literature valid for prognostic estimation of instability of “acute defects” in anterior GRF?

## Patients and methods

Patients that had a non-operatively treated GRF (“bankart-fracture” or “bony bankart lesion”, Ideberg 1B: ≥ 5 mm width of fragment [14]; according to Scheibel et al. [32]: Ib [solitary fragment] and Ic [multifragment]) were identified retrospectively. Small glenoid rim lesions (chip fractures, < 5 mm, Ideberg 1A) and fractures of the fossa glenoidalis (Ideberg 2–5 [14, 17, 23]) were excluded.

**Fig. 1:**
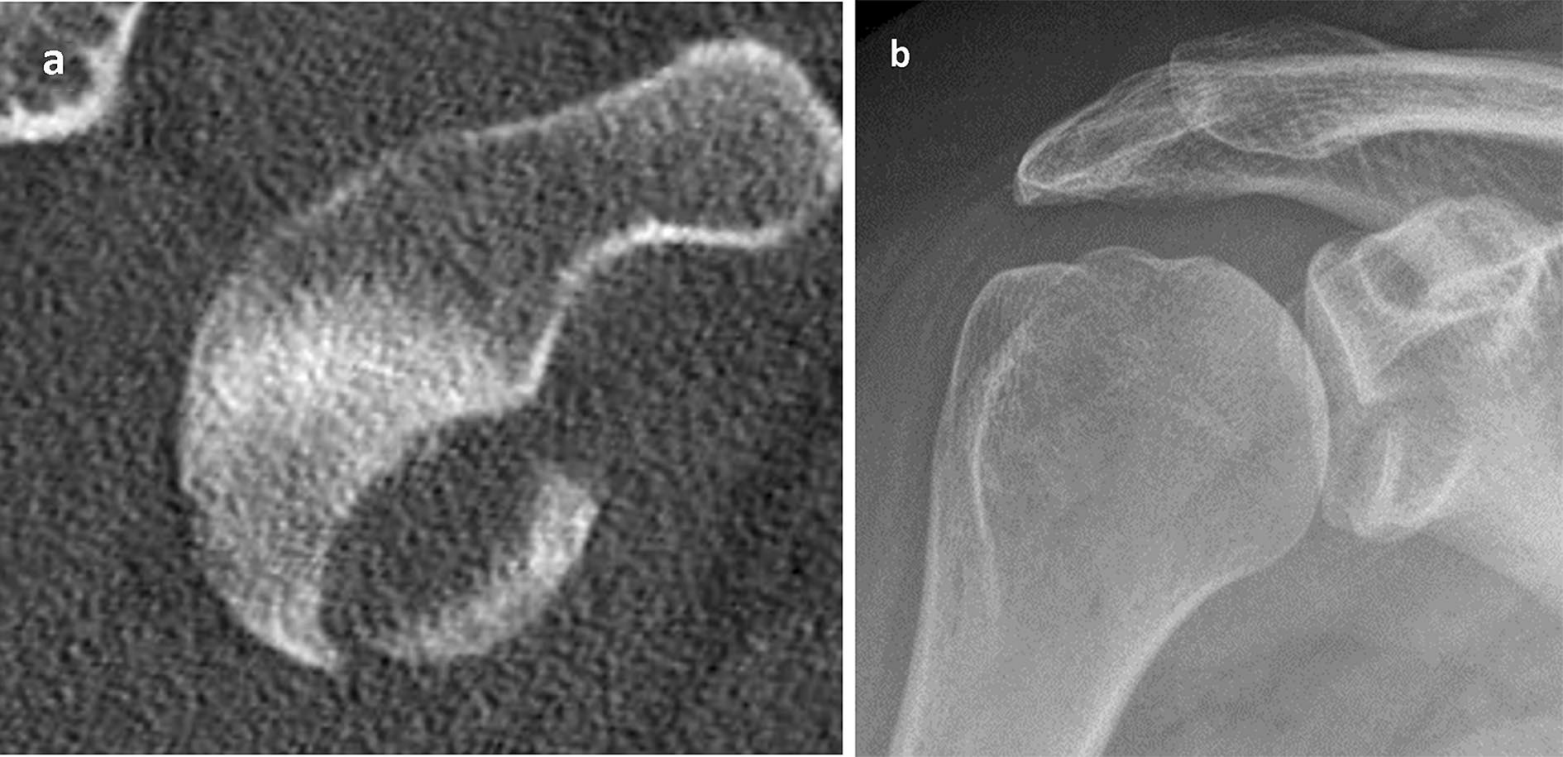
Concentrically reduced humeral head in the A.P.-radiograph is the main prerequisite for nonoperative treatment of GRF and represents the main criterion within radiographic controls (**b** CT en face view; **a** A.P.)

Indications for non-operative management included a fracture size of up to 1/3 of the glenoid surface even though there are no reliable data available regarding limits of fracture size. The concentricity of the humeral head in the anterior–posterior (A.P.) and axial radiograph or CT scan was the most important requirement for non-operative management (Fig. 1).

The degree of fragment dislocation was usually not a decisive criterion for either operative or non-operative treatment because no clear rules are provided within the literature. An exception are displaced fragments lateral to the glenoid plane into the inferior recessus, which was seen as an indication for surgery. Additionally, the general health of the respective patient was taken into account in the treatment decision and patients that declined an advised surgery were treated non-operatively.

### Clinical follow-up

The identified patients were invited to take part in the study after obtaining informed consent. The patients were questioned regarding their past history since the trauma with regard to redislocations, feelings of instability or any surgery of the affected shoulder. Then, the patients were examined clinically with a focus on instability and range of motion (ROM). The apprehension test at 90° abduction was used, the Western Ontario Shoulder Instability Index (WOSI) [7] and the age- and gender-matched Constant and Murley score (CMS) [6] were collected and were correlated with the size and the displacement of the glenoid fracture. Ethical approval for the study was given by the local ethical board.

**Fig. 2:**
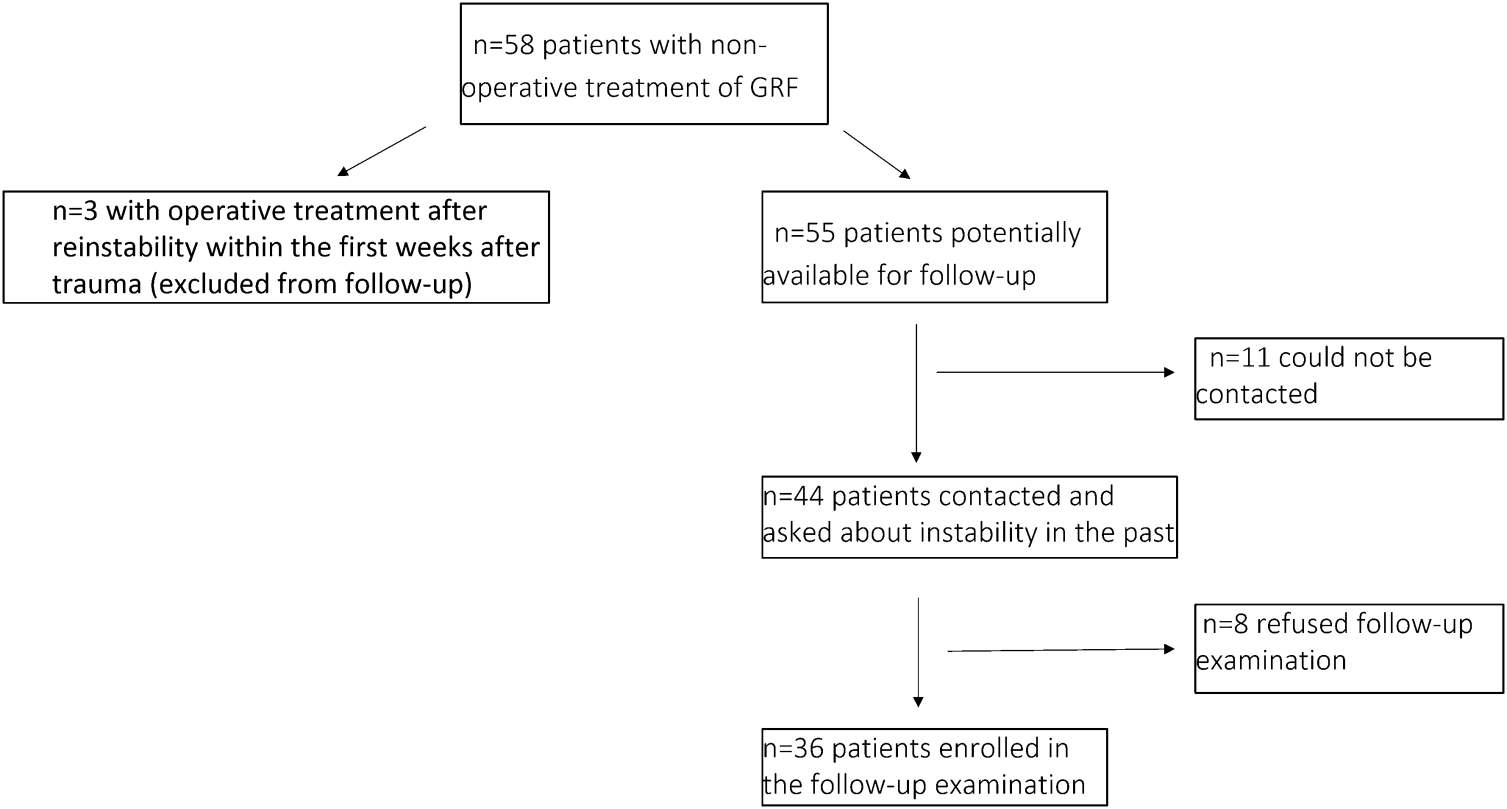
Flowchart of the non-operatively treated patients and inclusion in the study

From 1996 to 2013, *n* = 58 large Ideberg 1B fractures were initially treated non-operatively, of which *n* = 3 were treated operatively after re-instability and were excluded from the follow-up (remaining *n* = 55 potentially available for the study). There were *n* = 11 (20%) patients who could not be reached either because they deceased of unknown causes or the address had changed in the interim. Finally, *n* = 44 patients (80%) could be contacted successfully and could be asked about instability in the past since the initial trauma. Finally, *n* = 36 of those patients could be enrolled in the follow-up, because *n* = 8 patients did not want to come for the follow-up examination, but they indicated that the shoulder had remained stable since the initial trauma without any recurrence (Fig. 2).

The non-operative management included a sling in internal rotation in *n* = 32 patients for 1–3 weeks with pendulum exercises for 1 week and then free ROM. In *n* = 4 patients, the shoulder was immobilized in external rotation for three weeks.

### Radiographic analysis

In *n* = 30 (83%) of the followed-up patients, CT scan was available for analysis in addition to A.P. and axial radiographs (in *n* = 2 additional MRI was used). The fracture dimensions (length, width, etc.) and the morphology of the fracture line were captured with CT scan and divided into straight fracture line (72%) or uneven or rounded line (14%) (not definable in 14%). In patients with only conventional radiographs (*n* = 6, 17%), only the length and the medial displacement of the fragment could be measured with A.P.-radiographs, of which a good correlation with CT was shown by Maquieira et al. [21].

The trauma mechanism was differentiated between shoulder dislocation and only subluxation of the HH resulting in impression fractures without locking at the anterior glenoid rim. If the trauma mechanism could not be defined clearly within the database, or if there were no radiographs showing the HH dislocated, the radiographs and/or CT scan were analysed for Hill-Sachs lesions (HSL), which proves a previous complete locked dislocation of the HH. Current and most recent radiographs were analysed regarding healing of the fragment, osteoarthritis (classified according to Samilson/Prieto [31]) or possible chronical decentricity.

**Fig. 3:**
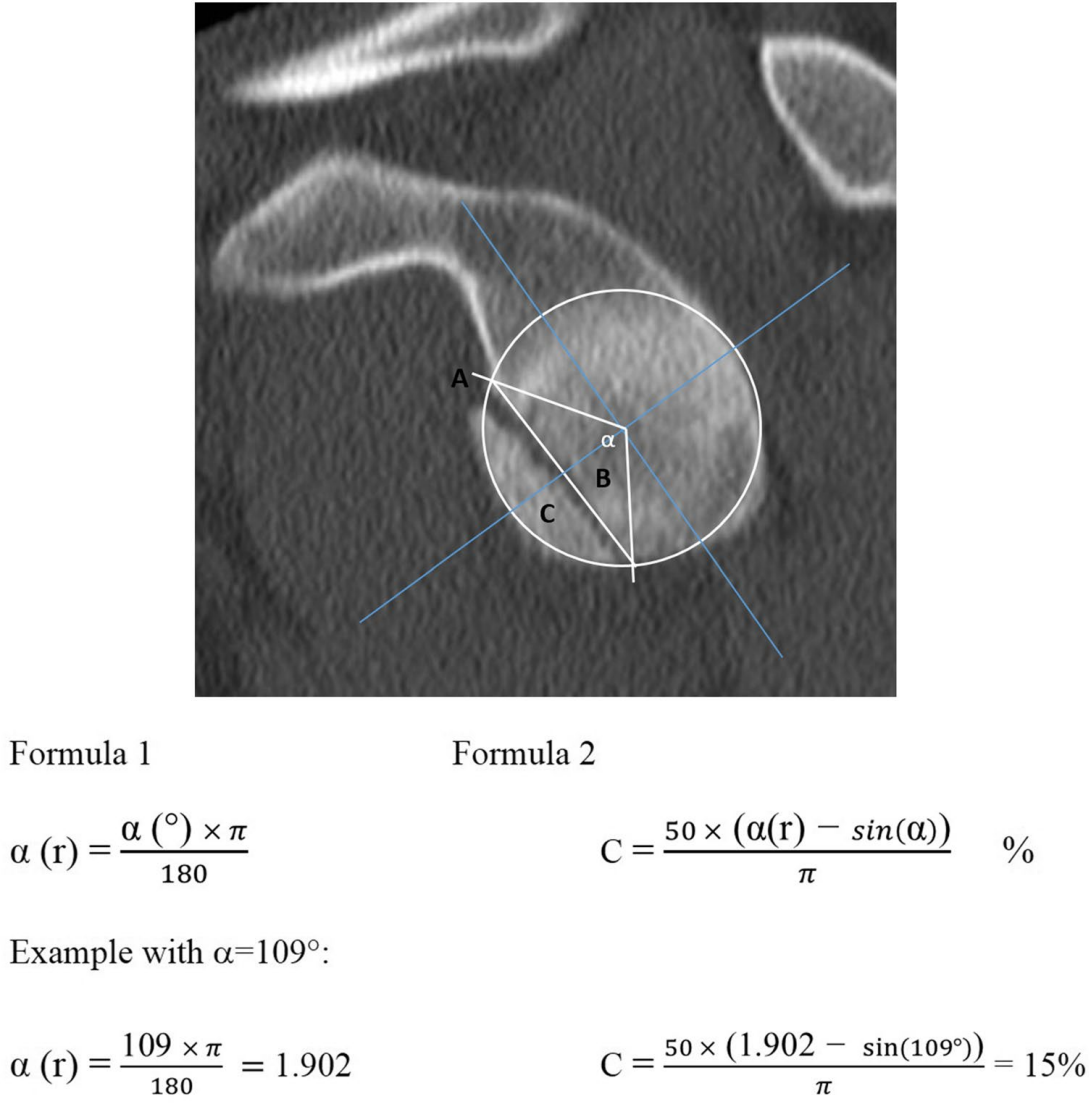
This segment A of the circle is the sum of a triangle B and the fracture C (A = B + C, see Figure). The area of C can be obtained by subtracting B from A, both of which are derived by elementary trigonometric theory. Finally, C is expressed as a percentage of the total circle size (see Formula and Figure). Calculation: *Formula 1* calculates the *radian* *α* (*r*) based on angle α in degrees (°). With *Formula 2***,** the percentage of glenoid surface fracture involvement (area C, Figure) can be calculated using *α* (*r*) (from Formula 1) and the sinus of the measured angle. An exemplary calculation of the GRF in Figure is presented below with a measured angle of 109° resulting in 15% glenoid fracture involvement (formula derived and recalculated from Wambacher et al. [37])

We critically revised the different methods for the CT scan for the glenoid surface in defect 3D-measurements [1, 5, 34] and 2D-measurements [1, 2, 10, 37] and descriptions of their respective advantages, whereas other authors reported no differences between 3 and 2D measurements [12, 19]. With the aim of obtaining the most exact calculation, we chose the technique of Wambacher et al., because of the advantage of measuring the surface within one exact and reproducible plane [37] (CT-2D en-face view),which is similar to the circle method of Baudi et al. [2]. In contrast to rounded fracture lines, a valid surface quantification is possible in cases with a straight or almost straight fracture line. The diameter of the contralateral (intact) glenoid serves as the reference for the affected side, and an identical circle with the same diameter was drawn on the glenoid surface. The measurements yield an angle “α”, which is then used for the simplified “formula” shown in Fig. 3.

In cases of either additional affections on the contralateral shoulder or deficient reconstructions (*n* = 10,28%) on the contralateral healthy side, the best fitting circle was drawn on the affected side on the posterior-inferior cortex of the glenoid. Two studies reported that the circle method could be used accurately without contralateral glenoid measurement [1, 12]. However, to verify these findings we tested the interobserver reliability for the circle method in cases without contralateral glenoid assessment using 12 stored thin-layer CT scans and created artificial defect sizes (15% and 30%) in the en-face view and two of the authors selected the best fitting circle at the posterior-inferior cortex in each glenoid (Siemens software Syngo.via VB10B, München, Germany). The intraobserver measurement revealed a significant correlation between the measurements for both of the investigators (first investigator: 15% defect: *r* = 0.98; 30% defect: *r* = 0.96; second investigator: 15% defect: *r* = 0.96; 30% defect: *r* = 0.90), and the interobserver correlation coefficient was also high (15% defect: *r* = 0.97; 30% defect: *r* = 0.93).

Additionally, the defect sizes were calculated using the 3D method of Itoi et al. [15] established for chronic glenoid erosions to verify whether these biomechanical results can be used for prognostic estimation of instability in acute fracture situations (Itoi et al. found that an osseous defect with a width of at least 21% of the glenoid length significantly decreases stability after only bankart repair). Additionally, these calculations are used for better comparison with the measurements within other studies dealing with GRF. The measurement is performed by en-face 3D-CT scan of the glenoid with a circle drawn with a diameter of the outer fitting circle of the glenoid and measurement of the distance from the outer circle to the fracture line (width in percent of the glenoid length) [32, 35].

### Statistical methods

The mean values, percentages and statistical differences were calculated, and statistical significance using the unpaired *t* test was defined as *p* < 0.05 (using SPSS statistical program® [SPSS Inc., Chicago, IL, 24.0]). The correlation coefficient was used including the Pearson test.

## Results

The average follow-up period of the *n* = 36 patients was 53 months (4.4 years; 12–140 month).

### Clinical follow-up

**Fig. 4:**
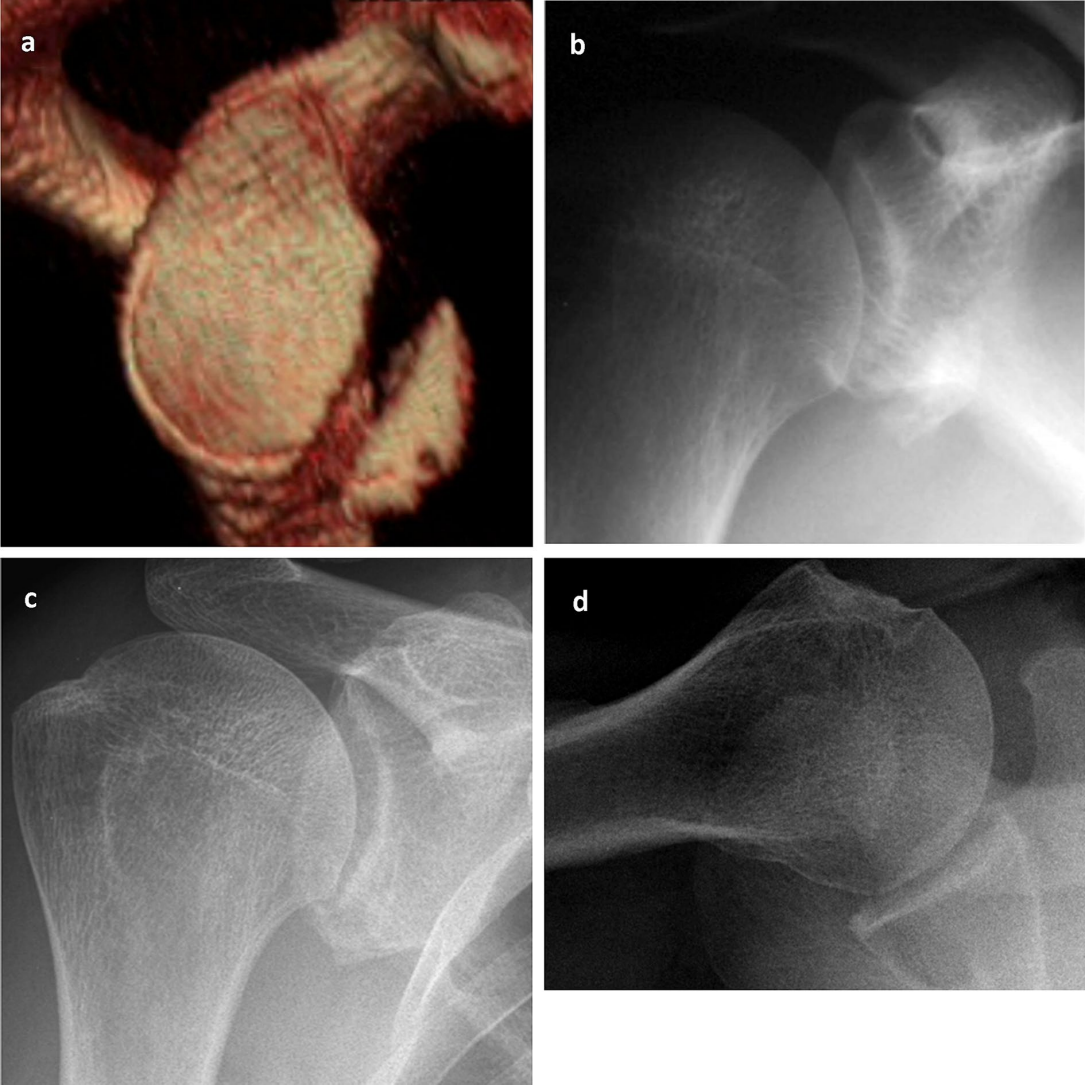
Non-operative treatment of an anterior GRF (**a** 3D-CT, enface view) in a 56-year-old patient after shoulder dislocation with a concentrically reduced humeral head (**b** A.P.-radiograph) without redislocation or signs of instability across the remaining course of 6 years and a healed fragment (**c**, **d** A.P. and axial-radiograph)

The a.-/g.-CMS was on average 93 points (± 11p, 61–100p), and the WOSI was on average 81% (± 22%, 35–100%) (Fig. 4, Clinical results see Table 1). The apprehension test was negative in all patients at final follow-up.

**Table 1:** Demographics and range of motion of patients after non-operatively treated anterior GRF (mean values, ranges, standard deviation and statistical difference)

Patient age (years)	Trauma: 58 ± 13 (33–86)	Follow-up: 62 ± 13 (35–87)	
Sex	Female: * n* = 19	Male: * n* = 17	
Side affected	Right: *n* = 27 (75%)	Left: * n* = 9 (25%)	
Non-/Dominant side affected	Dominant side: * n* = 25 (69%)	Non-dominant side: * n* = 11 (31%)	
a.-/g.-CMS	93 points (± 11 points, 61–100 points)		
WOSI	81% (± 22%, 35–100%)		
Range of motion	Affected side	Contralateral side	Statistical Difference
Flexion	169° ± 20 (120–180°)	175° ± 13 (120–180°)	*p* = 0.3
Abduction	163° ± 26 (90–180°)	173° ± 15 (110–180°)	*p* = 0.1
External rotation (0–10 points within Constant score: external rotation with the hand back of the head including elbow position)	9 ± 1 points (6–10)	9 ± 1 points (8–10)	*p* = 0.1
Internal rotation (0–10 points within Constant score: position of the hand on the back)	9 ± 2 points (6–10)	9 ± 0.5 points (8–10)	*p* = 0.2

*n* = 3 (8%) had operative interventions on their shoulder since the initial trauma. There was posttraumatic shoulder stiffness in two patients. One patient needed an arthrolysis 6 months after injury, and one patient suffered a polytrauma with additional injuries (e.g., fossa glenoidalis fracture at the opposite site) and presented with posttraumatic shoulder stiffness after prolonged ICU stay. In another patient with shoulder dislocation, persistent pain at the long head of the biceps tendon (LHB) 5 months after trauma led to an arthroscopy in which the glenoid fracture was found to be healed, but the labrum was ruptured, and an additional pulley lesion was detected. A tenodesis of the LHB was subsequently performed.

One additional patient (58 years) presented with painful but tolerable LHB at the examination 3 years after the trauma, but the duration of the pain could not be determined retrospectively. One patient described mild pain but with free ROM 5 years after trauma, and an antero-superior non-retracted tear of the supraspinatus tendon without fatty infiltration of the muscle (MRI) was found (treated by rotator cuff repair six years after trauma), whereas a direct correlation with the trauma 5 years ago remains unclear because of the lack of an initial MRI.

### Radiological analysis and correlations

The intraarticular step-off (medial displacement) was on average 4 mm (± 3 mm; 0–14 mm). The measurement within the en-face slices showed a glenoid fracture involvement of 21% (± 11%; 10–52%) on average. Derived from the measurements of Itoi et al. [15], the fractured surface involvement had a mean of 25% (± 6.6; 17–40%). The majority of the patients (*n* = 28; 78%) showed a non-dislocated or an already spontaneously reduced HH immediately after trauma. An HSL was found in most of the patients, whereas in only *n* = 7 patients (19%), no HSL could be detected, and all these shoulders were concentrically reduced in the initial radiographs (Table 2).

**Table 2:** Pathomorphology and fracture characteristics in *n* = 36 patients after non-operatively treated anterior GRF (mean values, ranges, standard deviation and statistical difference)

Averaged size of glenoid fracture	21% (± 11%; 10–52%)	
Averaged medial displacement of the (major) fragment (coronary plane)	4 mm (± 3 mm; 0–14 mm)	
Averaged displacement of the (major) fragment in the glenoid plane (sagittal)	3 mm (± 2 mm; 0–8 mm)	
Mean fragment length	24 mm (± 4 mm; 17–36 mm)	
Mean fragment width	9 mm (± 3 mm, 5–14 mm)	
Number of fragments	1 fragment:	*n* = 30 (83%)
	2 fragments:	*n* = 5 (14%)
	comminuted:	*n* = 1 (3%)
Hill-Sachs-lesion	Yes: * n* = 28 (78%)	No: * n* = 8 (22%)
Major tuberosity fracture	Yes: * n* = 5 (14%)	No: * n* = 31 (86%)
Position of humeral head at initial radiographs after trauma		
Locked humeral head at anterior glenoid		*n* = 7 (19%)
Non-dislocated or already spontaneously reduced		*n* = 28 (78%)
Subluxation		*n* = 1 (3%)

Practically no correlation was found between the a.-/g.-CMS and the degree of displacement (*r* = − 0.08; *p* = 0.6) and the fracture size (*r* = − 0.29; *p* = 0.2). Additionally, there was no correlation between the WOSI and the degree of displacement (*r* = − 0.14; *p* = 0.4) and the fracture size (*r* = − 0.37; *p* = 0.06) (Differentiation of fracture morphology and clinical results see Table 3).

**Table 3:** Differentiated correlation between displacement/ fractures sizes and clinical results of the patients (mean values, ranges, standard deviation and statistical difference)

	Non-displaced/ slightly displaced fragments (0–3 mm, mean 1.6 mm)	Widely displaced fragments (4–14 mm, mean 5.8 mm)	Statistical difference
Patients (*n*)	*n* = 19	*n* = 17	
Mean age	58 years	67 years	
a.-/g.-CMS	92 points ± 11	94 points ± 12	*p* = 0.7
WOSI	79% ± 20	81% ± 25	*p* = 0.8

	Fracture size ≤ 20% (10–20%; mean 16% ± 3)	Fracture size ≥ 21% (23–52%; mean 34% ± 12)	
Patients (*n*)	*n* = 19	*n* = 8	
Mean age	58 years	67 years	
a.-/g.-CMS	94 points ± 11	87 points ± 16	*p* = 0.2
WOSI	83% ± 17	69% ± 29	*p* = 0.2
Fragment displacement	Mean 3 mm ± 2, 0–7 mm	Mean 6 mm ± 3, 4–14 mm	*p* = 0.01

**Fig. 5:**
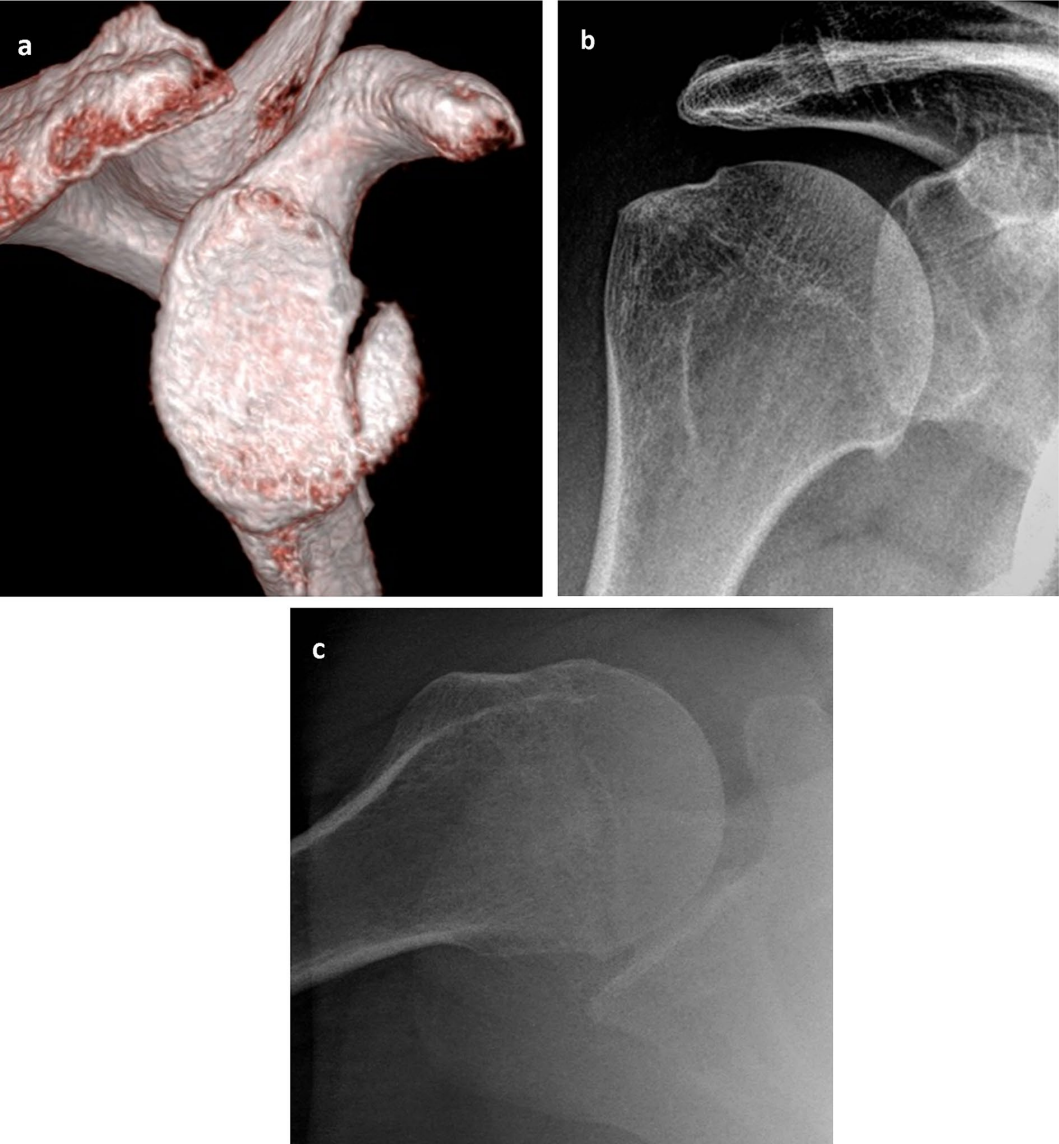
48-year-old patient with a minimally displaced non-operatively treated GRF (**a** 3D-CT, enface view) presented moderate OA (**b** A.P.-radiograph) 4 years later without reinstability and a free ROM (**b**, **c** A.P. and axial-radiograph)

OA, which had developed in the meantime, was found in *n* = 11 patients [*n* = 7 patients: grade II (*n* = 2 of these patients had already grade I at the time of trauma), *n* = 4: grade III] (Fig. 5). Three patients had pre-existing OA (grade I–II) within the radiographs after trauma without progression. In comparison between the patients with and without OA, the patients with OA were on average slightly older at the time of trauma (60 years vs. 56 years, *p* = 0.6) and showed slightly worse results in the clinical scores (a.-/g.-CMS: 88 vs. 93 points; WOSI: 73% vs. 82%).

### Reinstability

Of all primarily non-operative treated *n* = 58 patients, detected or reported re-instability could be found in *n* = 5 patients [fracture displacement on average 4 mm [± 2 mm], average age: 69 years]). There were redislocations found within radiographic controls in *n* = 2 patients (18% and 22% fracture size, 6 mm and 2 mm displacement), both within the first week after the trauma and these two patients were then treated surgically and excluded from the follow-up. In a further patient, a decentricity (or subluxation) of the HH was detected in the regular radiograph control after 3 days with subsequent operation (excluded from follow-up).

Within the followed-up patient cohort (*n* = 36), reinstability was retrospectively detected in two patients (6%) within the first days or weeks after trauma. In one of these patients decentricity was found after 14 days, but with continued non-operative treatment without detectable instability in the following years. Within the interview, the other patient reported redislocations within the first week after trauma with autonomous reduction. Surgery was then advised, but the patient refused, and the shoulder remained stable until follow-up within the examination.

All the patients with re-instability had an HSL, and none had a fracture of the major tuberosity.

## Discussion

The results demonstrate that fragment size or displacement is not correlated with instability in large anterior GRF within a mainly middle-aged patient group. The rate of recurrence after non-operative treatment is low, and all recurrence occurred within the acute or “vulnerable” period (within the first days and weeks) after trauma. Most patients showed very good to excellent clinical results. The present study is the largest followed-up cohort of non-operatively treated large anterior GRF, and a reproducible fracture quantification was performed for the first time in conservative glenoid fractures.

Ideberg was the first author describing non-operatively treated anterior GRF and found good to excellent results but no information to ROM or scores were given.[13]. Maquieira et al. published the first analysis of a patient group of non-operatively treated large GRF with measured displacement of the fragment in 14 patients and found excellent clinical results without reinstability [21], and another recently published trial (with 30 patients) detected excellent results with only one case of reinstability [38]. A further study confirmed those results in ten patients, and no recurrence or signs of clinical instability were reported [18]. Within these studies, no further complications or concomitant lesions were found [18, 21, 38].

Salomonsson et al. reviewed 12 cases of GRF and found that bony bankart was a positive predictive factor for stability [30].

Sugaya et al. [34] described the morphology of the anterior glenoid rim with a residual bone fragment in 50% of their recurrent cases. However, the size of the fragments is of importance in the assessment. Sugaya et al. reported only one fragment representing 26.9% of the glenoid (using 3D-measurement), whereas all other fragments were of medium size (10.6% on average) or small size (2.9% on average) with an overall average fragment size of 7.7% (!) within the patient group, which corresponds to small “chip fractures” in most of the cases. In contrast to that finding, the fragment size in our study was on average 21% (± 11%) using a similar quantification circle technique as described by Sugaya et al. Furthermore, in the study by Sugaya et al.[34], the average age of the patients with recurrence was 24 years, whereas the average age within our patient group was 58 years. Thus, in our eyes, the age of the patient group has to be considered for interpretation of the data because of the well-known fact that younger individuals have a higher risk for recurrence [22, 29, 36]. This finding is confirmed by the fact that the patient series of the above-mentioned studies of non-operative treated GRF had a mean age of 53 years [21], 57 years [18] and 48 years with only one case of instability [38].

Most chronic glenoid defects are not caused by large GRF but are the result of recurrent erosion [10, 22]. The biomechanical study of Itoi et al. [15] provided important findings for the understanding of these chronic defects (≥ 21% of bony defect significantly decreases stability) but can obviously not be generalized to acute glenoid defect situations, as  is sometimes brought into context within the literature [4, 24]. Because the measurement of defects related to the glenoid using the technique of Itoi et al. [15] showed a mean 25% defect size within our patient group, which would be expected to result in a significantly higher recurrence rate over several years (90% [!] of the patients with a glenoid fracture measured with this method had a defect ≥ 20% within our study). The “acute glenoid defect” represents another pathology, whereas hypothetically, the existing fragment (with its labral ring) may have a “bracing” effect in cases of non-displaced fractures. However, interestingly, no instability could be found in the apprehension test at the follow-up, even in widely displaced fragments, and the intraarticular displacement was the same within patient group without recurrence compared to the patients with recurrence (both 4 mm on average).

For the role of the soft tissue “stabilizers” (labrum, capsule, etc.), no statement can be given. Against this background, Plath et al. [25] reported in their series of arthroscopically refixed bony bankart lesions that a remaining glenoid bone deficiency or non-union did not influence the outcome, and Kim et al. [16] showed that, in small GRF, a soft tissue repair alone may be sufficient. Thus, the importance of the anterior bone fragment for stability remains unclear in acute GRF as well as the limits between operative and non-operative treatment, whereas both study groups treated patient cohorts with a lower average age (41 years [25] and 29 years [16]) in which reinstability may be rather expected, which makes surgical treatment reasonable.

A fracture of the greater tuberosity was not a predictive factor for reinstability in our study, which confirms the findings of other authors for general shoulder dislocations within a similar age group [11, 36]. Additionally, most of the patients (78%) in our study had an HSL, but all of the patients with a recurrence had an HSL, which shows that the patients with recurrence must have been locked at the anterior glenoid rim at least for seconds at the initial trauma. Next to shoulder dislocations, the underlying study shows that a direct impaction or impression of the glenoid without locking the HH seems to be a substantial but a rarer trauma mechanism shown by the lacking HSL and reduced HH in the initial radiographs in 22% of the cases.

Within the existing studies of non-operative treated GRF, mild osteoarthritis was found in one study in three (of 14) patients with grade I–II Samilson/Prieto (after a mean of 5.6 years) [21], within another study after 2 years in one (of 10 cases) (grade I Samilson/Prieto) [18], and a further study reported seven (of 30 cases) with OA (grad I–IV Samilson/Prieto) [38]. OA was also reported within studies of operative treatment of GRF. Within a study of an arthroscopically treated cohort of acute and chronic cases with a bony fragment, 70% full-thickness cartilage defects could be revealed after a mean of 82 months [25]. Another study of arthroscopic fixation of GRF with overall almost anatomic reconstruction of glenoid surface showed signs of OA in 7 of 23 cases, whereas the patients with OA within the cohort were 10 years older on average at the time of surgery [32]. Within our study, OA was found in 11 patients with slightly worse results in the clinical scores. Against this background, Edelson found in an anatomical analysis of 500 mature adult skeletons 27 specimens with GRF and HSL but without severe degenerative changes [8]. All those studies show that the development of OA in GRF is largely not yet understood.

The loss of follow-up is a limitation of the study, which is caused by the partially mid- and long-term follow-up period and the retrospective study design. The results are representative of middle-aged patients and no comprehensive statement can be given about younger patients (e.g. < 40 or < 30 years) in which non-operative therapy should be seen critically in terms of re-instability. An adequate interpretation of OA in relation to size and displacement of the fracture is not sufficiently possible, because of the different follow-up periods within the cohort. A further limitation is that *n* = 6 patients only underwent conventional radiographs initially, and no CT data after trauma were available for analysis. Finally, there is no control group presented here.

The general agreement for indication for non-operative treatment is a glenohumeral centricity in the A.P. view [9, 18, 21]. Interestingly, the degree of displacement of the GRF showed no difference in the clinical scores in the patient group. In contrast, the patients with very large (and more displaced) fragments showed slightly worse results in the a.-/g.-CMS and worse results in the WOSI score, but without significant difference. However, the patients with larger fractures were 9 years older on average, and it has been demonstrated, at least for the CMS in the literature, that age may influence the clinical outcomes [6]. Because of that fact and because of low statistical power in the group of advanced fracture sizes, an exact limitation of non-operative management cannot be given.

Acute or degenerative concomitant lesions (rotator cuff, LHB) were partially found within studies of arthroscopic or open refixation of the GRF [32, 33, 35]. Due to the fact that shoulder stiffness was found in two patients and symptomatic LHB pathology was detected in two cases, an additional MRI is recommended to exclude concomitant lesions, which could indicate surgical intervention.

Further studies with comparison between operative and non-operative treatment are necessary to more precisely qualify the limits of non-operative treatment of large anterior GRF.

## Conclusion

Most of the patients had good to excellent results after several years, which demonstrates that non-operative treatment is a successful alternative to operative management in middle-aged patients if there are no concomitant lesions in the recommended MRI and if the HH is concentrically reduced. The recurrence rate is low in these middle-aged patients but can occur within the subacute period (days/weeks) after trauma. Control radiographs are essential to avoid potential neglected decentricity or dislocation. Stability or re-instability did not depend on fracture size or displacement of the fragment. However, significantly larger anterior GRF sizes (with a displacement) should be evaluated critically for non-operative treatment but the limit of non-operative treatment remains unclear. Biomechanical trials of chronic glenoid deficiency are assessed as being not adequate for prognostic evaluation in terms of instability within the acute glenoid fracture situation.

## References

[1] Barchilon VS, Kotz E, Barchilon Ben-Av M, Glazer E, Nyska M (2008). A simple method for quantitative evaluation of the missing area of the anterior glenoid in anterior instability of the glenohumeral joint. Skeletal Radiol.

[2] Baudi P, Righi P, Bolognesi D (2005). How to identify and calculate glenoid bone deficit. La Chirurgia degli organi di movimento.

[3] Bigliani LU, Newton PM, Steinmann SP, Connor PM, McLlveen SJ (1998). Glenoid rim lesions associated with recurrent anterior dislocation of the shoulder. Am J Sports Med.

[4] Calvo E, Granizo JJ, Fernandez-Yruegas D (2005). Criteria for arthroscopic treatment of anterior instability of the shoulder: a prospective study. J Bone Jt Surg Br.

[5] Chuang TY, Adams CR, Burkhart SS (2008). Use of preoperative three-dimensional computed tomography to quantify glenoid bone loss in shoulder instability. Arthroscopy.

[6] Constant CR, Gerber C, Emery RJ, Sojbjerg JO, Gohlke F, Boileau P (2008). A review of the Constant score: modifications and guidelines for its use. J Shoulder Elbow Surg.

[7] Drerup S, Angst F, Griffin S, Flury MP, Simmen BR, Goldhahn J (2010). Western Ontario shoulder instability index (WOSI): translation and cross-cultural adaptation for use by German speakers. Orthopade.

[8] Edelson JG (1996). Bony changes of the glenoid as a consequence of shoulder instability. J Shoulder Elbow Surg.

[9] Goss TP, Owens BD, Iannotti JP, Williams GR (2006). Fractures of the scapula: diagnosis and treatment. Disorders of the shoulder: diagnosis and management.

[10] Griffith JF, Antonio GE, Yung PS (2008). Prevalence, pattern, and spectrum of glenoid bone loss in anterior shoulder dislocation: CT analysis of 218 patients. AJR Am J Roentgenol.

[11] Hovelius L, Eriksson K, Fredin H (1983). Recurrences after initial dislocation of the shoulder. Results of a prospective study of treatment. J Bone Jt Surg Am.

[12] Huijsmans PE, Haen PS, Kidd M, Dhert WJ, van der Hulst VP, Willems WJ (2007). Quantification of a glenoid defect with three-dimensional computed tomography and magnetic resonance imaging: a cadaveric study. J Shoulder Elbow Surg.

[13] Ideberg R, Bateman J, Welsh R (1984). Fractures of the scapula involving the glenoid fossa. Surgery of the shoulder.

[14] Ideberg R, Grevsten S, Larsson S (1995). Epidemiology of scapular fractures. Incidence and classification of 338 fractures. Acta Orthop Scand.

[15] Itoi E, Lee SB, Berglund LJ, Berge LL, An KN (2000). The effect of a glenoid defect on anteroinferior stability of the shoulder after Bankart repair: a cadaveric study. J Bone Joint Surg Am.

[16] Kim YK, Cho SH, Son WS, Moon SH (2014). Arthroscopic repair of small and medium-sized bony Bankart lesions. Am J Sports Med.

[17] Konigshausen M, Coulibaly MO, Nicolas V, Schildhauer TA, Seybold D (2016). Results of non-operative treatment of fractures of the glenoid fossa. The Bone Jt J.

[18] Kraus N, Gerhardt C, Haas N, Scheibel M (2010). Conservative therapy of antero-inferior glenoid fractures. Unfallchirurg.

[19] Magarelli N, Milano G, Baudi P (2012). Comparison between 2D and 3D computed tomography evaluation of glenoid bone defect in unilateral anterior gleno-humeral instability. Radiol Med (Torino).

[20] Maier D, Izadpanah K, Bayer J (2017). Influencing factors and complications in open treatment of acute anterior glenoid rim fractures. Unfallchirurg.

[21] Maquieira GJ, Espinosa N, Gerber C, Eid K (2007). Non-operative treatment of large anterior glenoid rim fractures after traumatic anterior dislocation of the shoulder. J Bone Jt Surg Br.

[22] Milano G, Grasso A, Russo A (2011). Analysis of risk factors for glenoid bone defect in anterior shoulder instability. Am J Sports Med.

[23] Nork SE, Barei DP, Gardner MJ, Schildhauer TA, Mayo KA, Benirschke SK (2008). Surgical exposure and fixation of displaced type IV, V, and VI glenoid fractures. J Orthop Trauma.

[24] Osti M, Gohm A, Benedetto KP (2009). Results of open reconstruction of anterior glenoid rim fractures following shoulder dislocation. Arch Orthop Trauma Surg.

[25] Plath JE, Feucht MJ, Bangoj R (2015). Arthroscopic suture anchor fixation of bony bankart lesions: clinical outcome, magnetic resonance imaging results, and return to sports. Arthroscopy.

[26] Porcellini G, Campi F, Paladini P (2002). Arthroscopic approach to acute bony Bankart lesion. Arthroscopy.

[27] Raiss P, Baumann F, Akbar M, Rickert M, Loew M (2009). Open screw fixation of large anterior glenoid rim fractures: mid- and long-term results in 29 patients. Knee Surg Sports Traumatol Arthrosc.

[28] Robinson CM, Kelly M, Wakefield AE (2002). Redislocation of the shoulder during the first six weeks after a primary anterior dislocation: risk factors and results of treatment. J Bone Jt Surg Am.

[29] Rowe CR (1956). Prognosis in dislocations of the shoulder. J Bone Jt Surg Am.

[30] Salomonsson B, von Heine A, Dahlborn M (2010). Bony Bankart is a positive predictive factor after primary shoulder dislocation. Knee Surg Sports Traumatol Arthrosc.

[31] Samilson RL, Prieto V (1983). Dislocation arthropathy of the shoulder. J Bone Jt Surg Am.

[32] Scheibel M, Hug K, Gerhardt C, Krueger D (2016). Arthroscopic reduction and fixation of large solitary and multifragmented anterior glenoid rim fractures. J Shoulder Elbow Surg.

[33] Scheibel M, Magosch P, Lichtenberg S, Habermeyer P (2004). Open reconstruction of anterior glenoid rim fractures. Knee Surg Sports Traumatol Arthrosc.

[34] Sugaya H, Moriishi J, Dohi M, Kon Y, Tsuchiya A (2003). Glenoid rim morphology in recurrent anterior glenohumeral instability. J Bone Jt Surg Am.

[35] Tauber M, Moursy M, Eppel M, Koller H, Resch H (2008). Arthroscopic screw fixation of large anterior glenoid fractures. Knee Surg Sports Traumatol Arthrosc.

[36] te Slaa RL, Wijffels MP, Brand R, Marti RK (2004). The prognosis following acute primary glenohumeral dislocation. J Bone Jt Surg Br.

[37] Wambacher MOJ, Rieger M München (2010, 117–123) Konventionelle Radiologie und Computertomographie der Schulter. In: Habermeyer P, Lichtenberg S, Magosch P: Schulterchirurgie

[38] Wieser K, Waltenspul M, Ernstbrunner L et al. (2020) Nonoperative treatment of anterior glenoid rim fractures after first-time traumatic anterior shoulder dislocation: a study with 9-year follow-up. JB JS open access 5(4) 10.2106/JBJS.OA.20.00133 PMC7757836 33376928

